# Veterans Traumatic Brain Injuries and Neurosurgical Challenges: A Narrative Review

**DOI:** 10.1002/hsr2.70863

**Published:** 2025-05-26

**Authors:** Ali Mortezaei, Negin Safari, Hediye Gholamshahi, Kimia Kazemzadeh, Abbas Tafakhori

**Affiliations:** ^1^ Network of Neurosurgery and Artificial Intelligence (NONAI) Universal Scientific Education and Research Network (USERN) Tehran Iran; ^2^ Student Research Committee Gonabad University of Medical Sciences Gonabad Iran; ^3^ School of Medicine Tehran University of Medical Sciences Tehran Iran; ^4^ Students Research Committee (SRC) Dezful University of Medical Sciences Dezful Iran; ^5^ Iranian Center of Neurological Research, Neuroscience Institute, Imam Khomeini Hospital Complex Tehran University of Medical Sciences Tehran Iran; ^6^ Department of Neurology, School of Medicine Tehran University of Medical Sciences Tehran Iran

**Keywords:** brain injury, neurosurgery, TBI, traumatic brain injury, veterans

## Abstract

**Background:**

Veterans experience a high prevalence of traumatic brain injuries (TBIs) due to combat‐related incidents such as explosive blasts, penetrating injuries, and blunt force trauma. These military TBIs are often more complex than civilian TBIs, leading to increased long‐term neurological consequences. Over 414,000 service members have been diagnosed with TBI since 2000, highlighting the need to understand the implications of these injuries and the neurosurgical challenges in their treatment.

**Methods:**

This narrative review examines the literature on TBIs in veterans, focusing on the characteristics, health impacts, and neurosurgical challenges associated with these injuries. The review synthesizes relevant articles to provide an overview of the topic.

**Results:**

Veterans with TBIs commonly experience cognitive deficits, including impairments in memory, executive functioning, processing speed, and visual disturbances. Research explores the relationship between TBI and neurodegenerative diseases, with some studies indicating a correlation between TBI severity and an increased risk of all‐cause and vascular dementia. Managing TBIs in veterans presents neurosurgical challenges such as timely diagnosis and intervention, tailored treatment approaches due to injury variability (blast vs. blunt trauma), and co‐occurring conditions like PTSD and depression. Initial medical measures include osmotherapy, sedation, hyperventilation, oxygenation, control of temperature and infection. In specific scenarios, an external ventricular drain (EVD) may be necessary to drain cerebrospinal fluid (CSF).

**Conclusion:**

Addressing TBIs in veterans necessitates a multidisciplinary approach with timely neurosurgical interventions, comprehensive rehabilitation, and mental health support. Future research should develop targeted treatments and explore novel technologies to improve recovery outcomes. Clinicians should prioritize early screening for TBI and co‐occurring conditions, while policymakers should improve access to specialized TBI care, ultimately enhancing veterans' long‐term quality of life.

## 1 Introduction

Traumatic brain injury (TBI) is a condition that occurs when the brain function is disrupted due to a traumatic event, resulting in memory impairment, loss of consciousness, changes in mental state, and/or neurological deficits [1]. TBI is considered the “signature injury” of modern military conflicts, and it is unclear whether military service members and veterans have different TBI outcomes compared to civilians due to differences in demographics, injury characteristics, or treatment received [2]. Every year in the United States, about 1.7 million people suffer from TBI, and more than 5 million of those hospitalized for TBI are currently living with a disability [3]. TBI can be caused by various events, including motor vehicle accidents, falls, assaults, head strikes, sports accidents, and blast injuries from military conflicts [4]. From the Afghanistan and Iraq conflicts alone, over 250,000 service members have been diagnosed with a TBI [5].

Mild traumatic brain injury (mTBI) is a significant concern among veterans of Operation Enduring Freedom (OEF)/Operation Iraqi Freedom (OIF)/Operation New Dawn, with prevalence rates ranging from 15% to 30% for those engaged in active combat in Afghanistan and Iraq [6, 7, 8]. Although most people recover well from mTBI [9], some continue to experience symptoms for months and even years after the injury [10, 11, 12, 13]. With over 200,000 incident diagnoses of mTBI in the military population since 2000 and an unspecified number of repeat concussions in the deployed population, many service members and veterans may continue to experience symptoms following mTBI. This presents a challenge to healthcare providers who treat them [14].

Neurosurgery plays an essential role in the management of TBI among military personnel, both in the acute stabilization of TBI during combat and the chronic rehabilitation process [15, 16]. Neurosurgeons can perform life‐saving procedures such as decompressive craniectomy which increases the chances of survival and rehabilitation for soldiers who have suffered severe head injuries [15]. However, even after successful acute management, the delayed consequences of TBI can be devastating, both medically and psychosocially [17].

This paper will explore the challenges that veterans with TBI face and the role of neurosurgery in their management. Addressing TBIs in veterans presents unique challenges compared to civilian populations due to the specific nature of injuries often sustained in military settings, such as blast‐related trauma and penetrating head injuries. These injuries may result in more complex and diffuse patterns of brain damage, leading to distinct clinical presentations and long‐term sequelae. Additionally, veterans often experience co‐occurring conditions such as posttraumatic stress disorder (PTSD), depression, and chronic pain, which can complicate TBI diagnosis and management. The combination of these factors necessitates tailored treatment approaches that consider the specific needs and circumstances of veterans with TBIs, highlighting the importance of specialized care and research focused on this population.

## 2 Methods

To conduct this narrative review, a comprehensive literature search was performed across PubMed/MEDLINE, Embase, Scopus, and Web of Science, focusing on studies related to traumatic brain injury (TBI) in veterans and associated neurosurgical challenges. The search strategy combined keywords and Medical Subject Headings (MeSH terms), including (“Traumatic Brain Injury” OR “TBI” OR “Brain Injuries”) AND (“Veterans” OR “Military Personnel” OR “Service Members”) AND (“Neurosurgery” OR “Surgical Management” OR “Treatment Outcomes”); “TBI in Veterans” AND (“Long‐term Effects” OR “Complications” OR “Neurosurgical Intervention”); “Blast‐Related TBI” AND “Veterans” AND (“Treatment” OR “Rehabilitation”); “cognitive deficits”, “mental health”, “PTSD”, and “Visual impairment.” The search was limited to English‐language articles published between January 1, 2000, and December 30, 2024. Studies of any design (clinical trials, observational studies, case reports, and review articles) were included if they addressed the epidemiology, pathophysiology, clinical presentation, neurosurgical interventions, or long‐term outcomes of TBI in the veteran population. Studies were excluded if they were not in English, did not specifically address TBI in veterans, focused solely on pediatric or civilian TBI populations without relevance to veterans, discussed only acute TBI management without longer‐term outcomes or neurosurgical considerations, or were purely animal studies, conference abstracts, editorials, or letters without original data. The relevance of selected articles was assessed based on their contribution to understanding the challenges faced by veterans with TBI and the role of neurosurgery in their care, with emphasis on identifying risk factors, mechanisms of injury, diagnostic modalities, treatment strategies, and potential preventive interventions. Reference lists of included papers were also manually searched for additional relevant studies. Given the narrative nature of this review, a formal assessment of study quality or risk of bias was not conducted; instead, the evidence was synthesized qualitatively to provide a comprehensive overview, organized thematically to address key aspects of TBI in veterans.

## 3 What Is TBI?

Traumatic brain injury (TBI), is an injury that affects the brain's functionality. TBI can happen in various forms of trauma, including blunt, penetrating, and compression traumas. Due to the battlefield conditions, soldiers are prone to this injury. In an observational study done in 2022, between 9% and 28% of service members who fought in the Afghanistan war had been subjected to TBI [18]; similar numbers have been found by other studies [19]. These numbers point out that TBI should be taken very seriously. There are two types of TBI; primary and secondary. Primary TBI happens as a result of direct trauma. Different types of mechanical damage to the brain can result in two kinds of primary injuries: focal and diffuse brain injuries. Research has shown that both kinds of injuries often co‐exist in patients with moderate to severe TBI [20]; however, diffuse axonal damage (DAI) is responsible for about 70% of TBI cases. Focal brain damage occurs due to cuts, pressure, and shock forces, in the closed head and penetrating TBI, which show signs of skull fracture and contusion at the center of the injury site (coup) [21]. The coup has a necrotic area of neuronal and glial cells with reduced blood flow, leading to the formation of epidural and subdural hematoma and intracerebral bleeding in specific brain layers. Another discoloration may appear in tissues opposite to or around the coup (contre‐coup) because of another impact that happens when the brain bounces back and hits the skull [21]. The severity of the injury can cause cognitive impairment, behavioral changes, and hemiparesis.

Unlike focal injury, diffuse brain injury is caused by noncontact forces of fast deceleration and acceleration, which injure cerebral brain tissues by shearing and stretching forces. The strong pulling forces harm neuronal axons, oligodendrocytes, and blood vessels, causing brain swelling and a lack of oxygen in brain tissues [22]. The main feature of diffuse TBI is widespread damage of axons mainly in subcortical and deep white matter tissue such as the brain stem and corpus callosum, which affects axonal transport and breaks down axonal cytoskeleton. These axonal damages can last for months after TBI, indicating a link with delayed secondary damage of bleeding, brain swelling, and edema [23]. The extent of axonal injury and neuronal loss determines the severity of TBI.

Secondary TBIs are delayed and long‐lasting effects that come after the primary injury and their main cause is belated inflammatory responses. They can last from hours to years. These effects are caused by several factors, such as excessive release of excitatory neurotransmitters, impaired mitochondrial function, increased production of reactive oxygen species (ROS), oxidation of lipids, inflammation of the nervous system, degeneration of axons, and cell death by apoptosis. The details of secondary injuries won't be discussed in this article due to our limitation in character count.

## 4 Characteristics of TBI in Veterans

TBI sequelae have been extensively studied in the military/veteran population. Moderate‐to‐severe TBI is associated with a wide array of sequelae, including neurocognitive deficits, seizures, post‐concussive symptoms, depression, aggressive behaviors, and psychosis [24]. Additionally, TBI is associated with numerous deleterious changes affecting the visual system, such as dysfunctions of the oculomotor and binocular vision systems, strabismus, visual acuity, visual fields, reading abilities, and dark adaptation. Other sensory dysfunctions following head injury have also been frequently documented, including auditory, vestibular, and chemosensory problems [25, 26, 27, 28, 29, 30].

The symptoms of TBI can be divided into three categories: physical, perception/sensation, and cognitive, behavioral, or mental. Check out Table 1. to see some of the characteristic signs and symptoms of TBI in veterans [24, 31, 32, 33, 34, 35, 36, 37, 38].

**Table 1:** Characteristic signs and symptoms of TBI in veterans.

Physical symptoms	Perception/sensation symptoms	Cognitive, behavioural, or mental symptoms
Fatigue	Dysfunctions of the oculomotor and binocular vision systems	Neurocognitive deficits
Headache	Strabismus	Seizures
Dizziness	Visual acuity	Post‐concussive symptoms
Sleep disturbances	Visual fields	Depression
Sensory dysfunction (e.g., auditory, visual, vestibular, and chemosensory problems)	Reading abilities	Aggressive behaviors
	Dark adaptation	Psychosis
	Auditory problems	PTSD symptoms (e.g., flashbacks or nightmares, avoidance of stimuli, increased anger, arousal, and hypervigilance)
		Affective, cognitive, and somatic complaints (e.g., headache, insomnia, fatigue, irritability, cognitive dysfunction, and chronic pain)
		Neuropsychiatric symptoms such as depression, apathy, or agitation
		Impairment in activities of daily living
		Cognitive decline in attention, memory, and language performance

Sensory dysfunction following head injury has been frequently documented. Auditory, visual, vestibular, and chemosensory problems are more commonly evidenced among those with a history of TBI, particularly those exposed to blast. While the relationship between blast exposure and injury to the auditory and visual systems is established in the current literature, the rapid change in pressure associated with blast is likely to affect the air‐ and liquid‐filled organs that characterize all sensory systems. Neurosensory deficits after TBIs can frequently lead to disability and are, therefore, important to properly diagnose and treat [25], [39].

TBI is often associated with various negative changes in the visual system. Some of the most commonly reported visual problems in people with TBI include dysfunctions of the oculomotor and binocular vision systems [26, 27, 28]. These dysfunctions can lead to deficits in fixation, pursuits, saccades, vergence, and accommodation. Additionally, studies have found that the prevalence of strabismus is high in TBI patients [29, 40]. Other areas of TBI‐related visual impairment include visual acuity (VA), visual fields (VFs), reading abilities, and dark adaptation [29, 30]. People with TBI often complain of visual disturbances, reading difficulties, diplopia, and light sensitivity [41, 42, 43].

Sleep‐related issues such as hypersomnia and insomnia are treated based on the specific sleep‐related complaint, using pharmacological treatments. Fatigue after TBIs is difficult to treat, but medications such as methylphenidate and modafinil have been used with some success [31]. Cognitive effects, such as neuropsychological performance, typically associated with non‐blast‐related mTBI generally resolve within several weeks to 3 months post‐injury [9, 44, 45, 46, 47]. Self‐reported affective, cognitive, and somatic complaints, known as postconcussive (PC) symptoms, resolve within 2 weeks following a sports‐related mTBI. However, elevated rates of symptom reports may persist in general adult samples 1 year post‐injury or longer [45]. Symptoms shared among mild TBI, PTSD, and depression include headache, insomnia, fatigue, irritability, cognitive dysfunction, and chronic pain, which can be considered independently existing clinical processes or as components of a complex syndrome [6, 48, 49, 50, 51, 52]. Certain mental health disorders are linked with mild TBI, including depression, substance abuse, self‐destructive behavior, and PTSD [14].

TBI is associated with short‐ and long‐term behavioral disturbances and cognitive decline [53, 54, 55]. Many long‐term symptoms related to PTSD and TBI can also be observed in individuals with Alzheimer's disease (AD). Overlapping symptoms include cognitive decline in attention, memory, and language performance, neuropsychiatric symptoms such as depression, apathy, or agitation, and impairment in activities of daily living [56, 57, 58, 59]. Numerous epidemiologic studies have linked TBI to AD. A history of TBI may be associated with an earlier onset of AD and the apolipoprotein E ε4 (APOE ε4) allele may worsen outcome [1]. Other possible long‐term consequences of TBI are the development of aging‐related Parkinson's disease, which co‐occurs commonly with AD, amyotrophic lateral sclerosis, and other neurodegenerative disorders that involve coincidental cerebrovascular disease pathology [38, 60]. PTSD is an anxiety disorder that develops in some individuals after exposure to traumatic stress. PTSD symptoms include flashbacks or nightmares and avoidance of stimuli; and increased anger, arousal, and hypervigilance. Although these symptoms abate, they can persist for years or even decades. The overall lifetime prevalence of PTSD in US combat veterans is estimated at 6% to 31%. The neuropathology underlying PTSD, separate from that associated with TBI, is completely unknown [33, 34, 35, 36, 37, 38].

## 5 Neurosurgical Challenges

Mechanical concussions cause primary brain injuries following a traumatic insult. These are predominantly irreversible and tend to manifest secondary physiological, cerebrovascular, and psychological changes. However, untreated patients may experience long‐term deleterious consequences, emphasizing the role of early discrimination and intervention in minimizing the risk of following complications. The expansion of intracranial contents subsequent to TBI caused by intraventricular or intracranial hemorrhage, cerebral edema, and hydrocephalus can culminate in brain herniation. In addition, these factors expose the brain to a precarious state, such as compartment syndrome and cerebral ischemia [61, 62, 63].

Despite promising advancements in surgical techniques, managing TBI patients always comes with challenges. The primary purpose of acute neurosurgical care is to mitigate the occurrence of secondary injuries, which are particularly prevalent among veterans owing to their unique circumstances. The organization of patients for care based on the severity of the damage is facilitated by the initial assessment performed using the Glasgow Coma Scale (GCS), a comprehensive examination, and the evaluation of the patient's premorbid status. Patients with severe TBI are treated following the Brain Trauma Foundation Guidelines for the Management of Severe TBI [64]. Mechanisms that cause TBI differ among veterans compared to others, each necessitating distinct approaches and care. A study assessed 1388 military veterans, of which 240 patients were included in their investigation. Approximately 50% of the participants indicated experiencing multiple head traumas. The predominant cause of injury observed in the study was blast 33.1% (*n* = 63), object hitting head 31.7 (*n* = 61), fall 13.5% (*n* = 26), vehicular accident or crash 8.7% (*n* = 17), and knocked out by another person 6.2% (*n* = 12) [65].

The cornerstone management is to regulate intracranial pressure (ICP) and to optimize cerebral perfusion pressure (CPP). Initial medical measures include osmotherapy utilizing mannitol or hypertonic saline, sedation, hyperventilation, oxygenation, and control of temperature and infection. In specific scenarios, an external ventricular drain (EVD) may be necessary to drain cerebrospinal fluid (CSF) [64, 66, 67]. Frequently elevated ICP usually heralds incurability with medical treatment where the decompressive craniectomy may be indicated [66]. However, low GCS, male gender, and computed tomography (CT) scan findings with midline shift and compressed/absent basal cisterns was associated with emergency surgical approaches [65, 67], the issue remains a subject of ongoing debate.

RESCUEicp (Trial of Decompressive Craniectomy for Traumatic Intracranial Hypertension), a randomized clinical trial (RCT), evaluated 408 patients with TBI and late refractory elevated ICP beyond 25 mmHg who were treated with decompressive craniectomy or received intensive medical care. At 12 months, the rates of mortality, vegetative state, lower severe disability, and upper severe disability in 194 patients in the surgical group vs. 179 patients in the medical group were 30.4% vs. 52.0%, 6.2% vs. 1.7%, 18.0% vs. 14.0%, and 13.4% vs. 3.9%, respectively. The incidence of moderate disability and favorable recovery exhibited comparable trends within both cohorts. Additionally, the study showed that surgical patients experienced lower elevated ICP than medical patients (median, 5.0 vs. 17.0 h) but had a higher proportion of adverse events (16.3% vs. 9.2%) [68]. In contrast, DECRA (Decompressive Craniectomy in Patients with Severe Traumatic Brain Injury) randomly allocated 155 patients with TBI and early refractory elevated ICP to surgical or medical therapies. They concluded that at 12 follow‐up times postoperation, decompressive craniectomy did not yield improved outcomes and augmented vegetative survivors [69]. Moreover, in combat and austere circumstances requiring precise timing, the performance of craniectomy before 5.33 h of trauma significantly mitigates postoperative mortality [70].

Although measures mentioned earlier often apply to those with severe TBI or in critical status, neurosurgeons should exert different approaches to patients with mild TBI. Patients may be addressed in various ways based on the clinical circumstances, CT scan findings, GCS, and other symptoms or signs and comorbidities [66]. ICP monitoring could be considered in patients with moderate to good GCS. If the ICP is augmented and prolonged despite the ventilator therapy, osmotherapy, temperature regulation, and positioning, the next step could be CSF diversion [64]. Patients manifested with cerebral contusion and acute subdural and epidural hematoma, with significant midline shift, focal neurological deficits, and increased hematoma volume and width, can be candidates for evacuation [64, 66, 67]. Following the completion of monitoring or surgical procedures, patients who exhibit stability are often eligible for discharge within 24 to 48 h [66, 67].

Altogether, the comprehensive management of veterans with TBI is contingent upon several factors: GCS score, clinical state, and the accessibility of transportation resources and neurosurgical facilities. These factors significantly influence the outcomes of TBI treatment, thereby underscoring the importance of strategic planning.

## 6 Long‐Term Outcome and Prognosis

TBI affects many functions in the brain both in the long and short term. Considering long‐term outcomes, we will discuss three major areas; quality of life and physiatrist health following TBI, memory and risk of dementia, and mortality rate.

### 6.1 Quality of Life

In a study by Jay Schulz‐Heik et al. [2], a follow‐up was conducted on 118 service members who had moderate to severe brain injury within the past 5–15 years. For occupational engagement, they found that 38% were unemployed, 10% were either volunteering or looking for work, and 52% were either employed or attending school. Fifty‐one percent of the studied population took a class after their injury and 24% completed a degree. Twenty‐nine percent of them who were single got married and 59% of those who were married got divorced. Twenty‐five percent live by themselves and 40% socialize outside their home once a week or else. Sity‐nine percent of the veterans report a disability status but 71% have no difficulty in their daily living activities. Gray et al. [71] found that functional independence scores were improved 8 years after the accident.

### 6.2 Memory and Risk of Dementia

TBI is considered to be a major risk factor for the development of neurodegenerative diseases including Alzheimer's disease (AD) and dementia [72]. Barnes et al. [73] conducted a case‐control study on veterans to evaluate the risk of developing dementia in those with TBI. She found that veterans with a history of TBI have a higher chance of developing dementia than other peers (6.1% vs. 2.6%, respectively). The adjusted hazard ratio for demographic and medical comorbidities were 2.36 (95% CI, 2.10–2.66) for mild TBI without LOC, 2.51 (95% CI, 2.29–2.76) for mild TBI with LOC, 3.19 (95% CI, 3.05–3.33) for mild TBI with LOC status unknown, and 3.77 (95% CI, 3.63–3.91) for moderate to severe TBI. Raza et al. [74] reported that both PTSD and TBI have a considerable effect on dementia development.

### 6.3 Mortality Rate

A study was done by Howard et al. [19] to evaluate mortality after TBI in veterans serving after September 11th, 2001. The study included 2,516,189 veterans who served after 9/11, of whom 17.5% had mild TBI and 3.0% had moderate to severe TBI; 30,564 deaths were reported in total. The death rates for these veterans were higher than the general US population after adjusting for age, and the rates rose with the severity of TBI. The study estimated that there were 3858 (95% CI, 1225–6490) more deaths than expected among all these veterans. Of these, an estimated 2285 (95% CI, 1637–2933) had mild TBI, and 1298 (95% CI, 1023–1572) had moderate to severe TBI. Veterans with moderate to severe TBI made up 33.6% of the total extra deaths, which was 11 times higher than the expected proportion. Byers et al. [75] found that Veterans with mild and moderate‐to‐severe TBI had a higher risk of future death over the short term with a slight decrease after 6 months, but mild TBI cases had a lower hazard ratio which was constant over time. Leading causes of death were unintentional injury, stroke, and suicide which showed differences in TBI severity.

See Table 2 to find the summarization of key findings and limitations of included studies in our narrative review.

**Table 2:** Summarization of key findings and limitations of included studies.

Author	Reference	Design	Year	Key Findings	Major Limitations
McFall et al.	[33]	Cross‐sectional survey of 489 male veterans in VA substance‐abuse treatment	1991	− 10.7% overall (19.5% in Vietnam‐era) met criteria for combat‐related PTSD symptoms. − High combat exposure correlated with more psychiatric & adjustment issues (e.g., suicidality).	− Relied solely on the Mississippi PTSD Scale, no structured interview. − Substance‐abusing treatment seekers hamper broader generalizability. − Cross‐sectional design hamper cause inference.
Lew et al.	[29]	Descriptive observational study of a newly developed VA Polytrauma Network Site (PNS) clinic	2007	− Among 62 patients evaluated, high prevalence of post‐concussive symptoms, PTSD, sensory impairments, and cognitive deficits. < br/> ‐ Interdisciplinary approach improved patient satisfaction.	− Single‐site, small sample. < br/> ‐ No control group for comparison. < br/> ‐ Findings might evolve as the clinic matures.
Stelmack et al.	[28]	Retrospective EMR review in a VA polytrauma setting	2009	− Found frequent visual dysfunction (e.g., ocular motility, binocular vision) among TBI patients. − Highlighted importance of routine vision assessments in polytrauma care.	− Single‐site, retrospective approach. − Potential selection bias and no healthy comparison group.
Brahm et al.	[43]	Retrospective analysis of vision‐screening data from two VA programs	2009	− Inpatient cohort (moderate/severe TBI) had near‐normal acuity but higher incidence of visual field defects. − Outpatient cohort (mild TBI) showed convergence/accommodative deficits, strongly tied to blast exposure.	− Retrospective design with possible underreporting of mild deficits. − Variation in clinical examinations hamper consistent data.
Richardson et al.	[37]	Critical/narrative review of combat‐related PTSD prevalence	2009	− Summarized prevalence estimates for PTSD among US veterans ranging from 2%–17% (point) and 6%–31% (lifetime). − Highlighted wide methodological heterogeniety among studies.	− Narrative review lacks systematic quantitative synthesis. − Differences in sampling/measurement hamper direct comparability of results.
Smith et al.	[36]	Large population‐based prospective cohort (n > 150,000)	2001–2008	− Tracked mental/physical health in active, Reserve, and National Guard personnel, ~50% of whom deployed to Iraq/Afghanistan. − Connected specific deployment exposures with adverse outcomes over time.	− Heavily reliant on self‐report data, which can introduce recall bias. − 3‐year intervals might miss short‐term fluctuations.
Dougherty et al.	[42]	Retrospective cohort study of blast‐related TBI	2011	− 8.9% of blast‐TBI service members had ocular/visual disorder within 1 year. − Odds of visual dysfunction increased with TBI severity. − Conjunctival disorders more common in TBI vs. other injuries.	− Retrospective approach may underestimate true prevalence. − Excluded those with direct eye injuries, limiting generalizability.
Scholten et al.	[49]	Observational study of 55,070 VHA patients completing TBI evaluations	2012	− Moderate/severe neurobehavioral symptoms were common in those with TBI. − Most symptoms attributed to both TBI and co‐occurring mental health issues.	− Retrospective data extraction from clinical notes could be incomplete. − Sample is restricted to Veterans receiving VHA care.
Stricker et al.	[53]	Cross‐sectional comparison of OEF/OIF/OND service members (PTSD vs. non‐PTSD)	2016	− PTSD group exhibited significantly higher rates of memory impairment; no difference in attention or executive function. − Having comorbid depression did not alter PTSD group's cognitive findings.	− Non–treatment‐seeking sample with no substance abusers may hamper generilizability. − Modest sample sizes reduce power to detect smaller effects.
Schulz‐Heik et al.	[2]	Cross‐sectional telephone interviews of veterans 5–16 years post‐TBI	2016	− 52% employed or in school; 62% reported life satisfaction equal/better than pre‐injury. − Duration of Posttraumatic amnesia correlated with poorer long‐term outcomes.	− High attrition (38% unreachable). − Self‐reported data subject to potential recall bias. − Variation in rehab exposure not captured.
Lindquist et al.	[65]	Cross‐sectional survey of a national random sample of post–9/11 veterans	2017	− 17.3% met TBI criteria, half of which had multiple TBIs. − Mechanisms: blast 33.1%, object impact 31.7%, falls 13.5%. − Preservice TBI doubled odds of in‐service TBI.	− Entirely self‐report data may be inaccurate. − Only “worst” head injury measured. − Focused on veterans engaged with VA care.
Gray et al.	[71]	Retrospective review + prospective follow‐up in 44 TBI patients	2017	− Significant functional gains observed by discharge and maintained at 3‐month and 8‐year follow‐up. − Over 50% employed or continuing education after 8 years.	− Single‐cohort from one fiscal year, potential attrition bias. − Reliance on self‐report for long‐term outcomes.
Swana et al.	[25]	Retrospective cohort using DoD & VA administrative data (n = 570,248)	2018	− ~23% of Post–9/11 Veterans had ≥ 1 sensory dysfunction diagnosis; TBI strongly linked to auditory, visual, vestibular, chemosensory deficits. − Blast exposure especially implicated.	− Using ICD‐9‐CM codes can miss subclinical deficits. − Retrospective approach hamper direct cause‐effect inference.
Shackelford et al.	[70]	Retrospective observational analysis in 486 severe combat‐related TBI cases	2018	− Earlier decompressive craniectomy (within 5.33 h of injury) was associated with significantly lower mortality (adjusted HR 0.28). − Prehospital delays had greater impact on outcomes than in‐hospital delays.	− Only 44% had complete date/time data. − No long‐term functional metrics. − Retrospective design excludes those who died presurgery.
Barnes et al.	[73]	Retrospective cohort with propensity‐matched veterans (mild/moderate/severe TBI vs. non‐TBI)	2018	− Even mild TBI (no LOC) linked to ~twofold higher dementia risk. − Dementia risk escalated with TBI severity, suggesting possible long‐term neurodegenerative sequelae.	− Dependent on VHA medical records; early cognitive decline may be underdiagnosed. − No standardization for repeated TBIs.
Bhatnagar et al.	[31]	Narrative review of neurosensory deficits post‐TBI in polytrauma veterans	2019	− Summarized headache, vestibular, auditory, and visual problems as major contributors to persisting disability. − Advocated targeted interventions for improved functional recovery.	− Narrative approach may omit some relevant studies. − Heterogeneous data hamper uniform conclusions about best interventions.
Elder et al.	[72]	Review exploring TBI, PTSD, Alzheimer's, and chronic traumatic encephalopathy (CTE)	2019	− TBI predisposes to chronic inflammation relevant in Alzheimer's and CTE pathogenesis. − Blast‐related trauma specifically implicated in persistent mental health and neurodegenerative risk.	− No consensus criteria for diagnosing CTE. − Overlap in symptom profiles for PTSD/TBI hamper distinct evaluation.
Raza et al.	[74]	Narrative review on dementia risk factors (TBI, PTSD, sleep) in military populations	2021	− Moderate/severe TBI and PTSD both elevate dementia risk. − Cumulative or repeated TBIs may accelerate cognitive decline. − Identified poor sleep as another exacerbating factor.	− Most studies lack direct correlation between deployment and long‐term dementia. − Limited longitudinal data specifically examining sleep's role.
Byers et al.	[75]	Cohort study investigating mortality risk in veterans with mild or moderate‐severe TBI	2022	− Both TBI groups had increased early post‐injury mortality, highest in the first 6 months. − Leading causes of death varied by TBI severity (injury, stroke, suicide).	− Largely male sample from VA records. − TBI severity classification possibly inaccurate. − Observational design hamper definitive causal statements.

Abbreviations: DoD, department of defense; EMR, electronic medical record; HR, hazard ratio; ICD‐9‐CM, international classification of diseases, ninth revision, clinical modification; LOC, loss of consciousness; OEF/OIF/OND, Operation Enduring Freedom/Operation Iraqi Freedom/Operation New Dawn; PTSD, post‐posttraumatic stress disorder; TBI, traumatic brain injury; VA, veterans affairs; VHA, veterans health administration.

## 7 Emerging Methods

Emerging methods in TBI research are paving the way for innovative therapies aimed at improving recovery outcomes. One promising approach is optogenetics, which employs light‐sensitive proteins to control genetically modified neurons with high spatial and temporal precision. This technique has demonstrated potential in modulating neuronal activity and promoting recovery after TBI, as highlighted in recent reviews [76, 77]. Optogenetics builds upon advancements in bioengineering, optics, and genetics, enabling selective activation or inhibition of neuronal subpopulations and fostering neurogenesis through targeted stimulation [77]. The development of safer and more efficient light delivery methods, such as near‐infrared light, microwaves, two‐photon excitation, and magnetic resonant coupling, could significantly enhance the safety and efficacy of optogenetic tools. Additionally, incorporating cooling mechanisms into light delivery devices may mitigate thermal toxicity, optimize light transmission efficiency, and reduce tissue exposure time, which are critical for minimizing damage during treatment. These innovations represent a significant step forward in targeted neural repair [77]. Further advancements in optogenetics include noninvasive approaches like transcranial optogenetic stimulation using infrared wavelengths that penetrate deeper into brain tissue. These methods aim to overcome the invasive nature of current procedures while maintaining precision. Combining optogenetics with other therapies such as deep brain stimulation (DBS) or transcranial magnetic stimulation (TMS) could offer synergistic effects by enhancing specificity and reducing off‐target impacts [76, 77]. In parallel, multidisciplinary initiatives like TRACK‐TBI are revolutionizing TBI research by integrating clinical, imaging, proteomic, genomic, and outcome biomarkers to refine diagnostics and therapeutic strategies. These efforts aim to establish a comprehensive TBI Information Commons to support research and improve patient stratification for clinical trials. Such collaborative approaches underscore the importance of precision medicine in addressing the complex pathophysiology of TBI [78].

## 8 Gaps and Future Directions

A critical area requiring further investigation is the nuanced relationship between TBI severity, particularly mild TBI, and the subsequent risk of neurodegenerative diseases. While some studies suggest a correlation between TBI and increased risk of dementia, particularly vascular dementia and earlier onset Alzheimer's disease, the specific mechanisms driving this association remain unclear. Longitudinal studies with large cohorts of veterans, employing advanced neuroimaging techniques and biomarkers, are needed to elucidate the pathological processes linking TBI to neurodegeneration. Specifically, research should focus on identifying early predictors of cognitive decline and exploring potential protective factors. Furthermore, there is a need for studies that directly compare outcomes of TBI sustained through different mechanisms (e.g., blast vs. blunt force trauma) to identify any distinctive neuropathological trajectories and inform tailored treatment strategies. Understanding the interaction between TBI, PTSD, and other co‐occurring mental health conditions is also crucial, as these comorbidities can significantly impact recovery and long‐term well‐being. Future research should prioritize the development and validation of novel neurosurgical approaches and rehabilitation strategies specifically tailored to the unique needs of veterans with TBI. This includes exploring the potential of emerging technologies, such as minimally invasive surgical techniques, neuromodulation, and advanced neurorehabilitation programs, to improve functional outcomes and quality of life. Moreover, there is a critical need for research focused on optimizing the timing and intensity of interventions across the continuum of care, from acute management to long‐term rehabilitation. Studies should also investigate the effectiveness of multidisciplinary approaches that integrate neurosurgical care with mental health services, cognitive rehabilitation, and social support, to address the complex needs of veterans with TBI. Finally, research is needed to address the disparities in access to care and outcomes among different subgroups of veterans, particularly those in rural areas or with limited resources, to ensure equitable access to high‐quality TBI care [50, 51, 52].

## 9 Conclusion

In conclusion, addressing traumatic brain injuries in veterans requires a multifaceted approach that encompasses timely diagnosis, innovative neurosurgical interventions, and comprehensive rehabilitation strategies. The concept of innovative neurosurgical interventions encompasses advanced techniques such as minimally invasive surgery, stereotactic procedures, and the use of neuromodulation to optimize outcomes following TBI. These interventions aim to reduce secondary damage, improve cerebral blood flow, and enhance neuroplasticity. Comprehensive rehabilitation strategies involve a multidisciplinary approach, integrating physical therapy, occupational therapy, speech therapy, and cognitive rehabilitation to address the diverse needs of veterans with TBI. These strategies are tailored to improve motor skills, cognitive function, communication abilities, and overall independence, with the goal of maximizing functional recovery and enhancing quality of life.

The unique challenges posed by combat‐related TBIs necessitate collaboration among healthcare professionals to ensure holistic care. As research advances, it is crucial to develop targeted treatment protocols that address both the neurological and psychological needs of veterans. To translate these insights into actionable improvements, several key steps can be taken. Clinically, healthcare providers should prioritize early screening for TBI and co‐occurring mental health conditions, ensuring seamless integration of neurosurgical care with psychological support services. Policymakers can support this effort by allocating resources to enhance access to specialized TBI care for veterans in underserved areas. Additionally, future research should focus on developing evidence‐based guidelines for the optimal timing and intensity of neurosurgical interventions, as well as exploring novel technologies and therapies that can enhance recovery outcomes. By fostering a collaborative environment between researchers, clinicians, and policymakers, we can better address the complex needs of veterans with TBI and improve their long‐term quality of life.

## Author Contributions

**Ali Mortezaei:** investigation, writing – original draft. **Negin Safari:** investigation, writing – original draft. **Hediye Gholamshahi:** investigation, writing – original draft. **Kimia Kazemzadeh:** writing – review and editing, supervision. **Abbas Tafakhori:** validation, supervision.

## Disclosure

Corresponding had full access to all of the data in this study and takes complete responsibility for the integrity of the data and the accuracy of the data analysis. The authors declare that they have prepared this article in their “personal capacity” (in other words, “not as an official representative or otherwise on behalf of a sanctioned government”). All authors have read and approved the final version of the manuscript.

## Ethics Statement

The authors have nothing to report.

## Consent

The authors have nothing to report.

## Conflicts of Interest

The authors declare no conflicts of interest.

## Transparency Statement

The lead author Abbas Tafakhori affirms that this manuscript is an honest, accurate, and transparent account of the study being reported; that no important aspects of the study have been omitted; and that any discrepancies from the study as planned (and, if relevant, registered) have been explained.

## Data Availability

The authors have nothing to report.
